# Characterization of the oral virome in patients with diabetes mellitus

**DOI:** 10.3389/fcimb.2025.1607798

**Published:** 2025-09-03

**Authors:** Yidi Zhang, Yue Zhang, Guorui Xing, Ting Mei, Minhui Wang, Chunxia Huang, Hanzhi Yi, Yu Zhan, Sen Yang, Qiulong Yan, Shenghui Li, Changming Chen

**Affiliations:** ^1^ Guizhou University of Traditional Chinese Medicine, Guiyang, Guizhou,, China; ^2^ Puensum Genetech Institute, Wuhan, China; ^3^ The Fifth Affiliated Hospital of Southern Medical University, Guangzhou, China; ^4^ Department of Microbiology, College of Basic Medical Sciences, Dalian Medical University, Dalian, China; ^5^ iDepartment of Bochemistry and Molecular Biology, College of Basic Medical Sciences, Dalian Medical University, Dalian, China; ^6^ Department of Rheumatology and Immunology, the Second Affiliated Hospital of Guizhou University of Traditional Chinese Medicine, Guiyang, Guizhou, China; ^7^ Chongqing Precision Medical Industry Technology Research Institute, Chongqing, China

**Keywords:** oral viruses, diabetes mellitus, viral diversity, viral function, host

## Abstract

**Introduction:**

Diabetes mellitus (DM), a globally prevalent chronic metabolic disorder characterized by persistent hyperglycemia, has been increasingly linked to dysbiosis of the oral microbiome. However, the relationship between the virome, a crucial component of the oral microbiome, and DM remains poorly understood.

**Methods:**

To explore the characteristics of the oral virome in DM patients, we analyze the oral viral communities of 45 DM patients and 40 healthy controls (HC) using a publicly available metagenomic dataset.

**Results:**

Our analysis revealed no significant differences in a-diversity between DM patients and HC. However, *Podovirus* was enriched in DM patients, whereas *Microviridae* was more prevalent in HC. A total of 1,131 virus signal was identified, primarily belonging to the *Siphovirus* and *Myovirus* taxa. Notably, HC-enriched vOTUs exhibited broader host tropism, predominantly infecting *Prevotella*, *Fusobacterium*, and *Gemella*, whereas DM-enriched vOTUs showed narrower specificity for *Pauljensenia* and *Veillonella*. Cross-kingdom network analysis suggested that certain viruses (HMP_1157.k81_309051) may have potential links to the development of DM, and the bacteria genus F0040 might play a significant role in maintaining oral health. Additionally, the random forest model based on viral markers effectively distinguished between HC and DM patients (AUC =90.8%), significantly outperforming the bacterial model.

**Discussion:**

This indicates that these unique viral markers could serve as potential targets for DM intervention. Taken together, our findings reveal distinct alterations in the oral virome of DM patients and highlight its promise as a novel diagnostic and therapeutic target in metabolic disease research.

## Introduction

Diabetes mellitus (DM) not only compromises patients’ quality of life but also induces long-term damage, dysfunction, and failure in ocular, renal, neural, cardiac, and vascular systems (American Diabetes Association, 2011). With a global prevalence exceeding 820 million adults and projections estimating a rise to 1.31 billion by 2050, DM has emerged as a major chronic disease threatening public health (GBD 2021 Diabetes Collaborators, 2023). While its pathogenesis ​is influenced by both environmental and genetic factors, recent studies ​highlight the significant role of the gut microbiota in diabetes onset and progression.

However, the oral microbiota, as the second-largest microbial community in the body, also plays a crucial role in health maintenance (Sabharwal et al., 2019; Agho et al., 2021). In diabetic patients, the oral cavity shows increased levels of Lactobacillus, Corynebacterium, and *Pseudomonas*, while *Porphyromonas*, *Treponema*, *Prevotella*, and *Parvimonas* are reduced (Ganesan et al., 2017). Additionally, the prevalence of Candida albicans-induced oral candidiasis in diabetic patients is as high as 30%, further underscoring microbial dysbiosis (Karina et al., 2025). Although research traditionally focused on oral bacteria and fungi, yet oral viruses, despite being a key component of the oral microbiome, have received limited attention. Investigating the role of oral viruses in DM pathogenesis may provide novel insights into disease mechanisms and therapeutic opportunities.

The oral virome encompasses the entire viral community in the oral cavity, including bacteriophages, eukaryotic viruses, and free viral genetic material (Morrison et al., 2023). Metagenomic analyses indicate that bacteriophages constitute the dominant viral population in healthy oral ecosystems (Liang and Bushman, 2021; Baker et al., 2024). Under specific conditions, Mechanistically, these viral agents might promote pro-inflammatory cytokine production (e.g., IFN-γ, IL - 12, and TNF-γ) and oxidative stress, initiating inflammatory cascades that worsen systemic inflammation in diabetic individuals and potentially impair pancreatic islet function (Wang et al., 2018; Li et al., 2023; Lv et al., 2024). Beyond direct inflammatory effects, oral viruses may contribute to diabetes pathogenesis by altering microbial communities in both oral and gastrointestinal environments. Currently, research on the oral virome in DM patients remains limited. Considering the documented alterations in oral bacteriome composition among DM patients and the intricate virome-bacteriome interplay, comprehensive investigation of the oral virome in DM holds substantial scientific merit.

Recent technological advancements, particularly in high-throughput sequencing, have significantly enhanced our ability to characterize the complexity of the human virome. In light of growing interest in the DM virome, we analyzed publicly available deep shotgun metagenomic sequencing data from 45 DM patients and 40 HC to characterize the oral virome in DM (Heintz-Buschart et al., 2016). Through comprehensive reanalysis, we recovered over 35,000 non-redundant viral sequences and performed an in-depth examination of the oral virome. Our findings reveal substantial differences in oral viral community composition between DM patients and HC, with 1,131 non-redundant viruses showing significant abundance variations between the two groups. Additionally, our findings highlight complex interactions between oral viruses and bacterial populations, with the classification potential of these signatures evaluated in distinguishing DM patients from HC. This research provides new insights into potential mechanisms underlying DM pathogenesis and may help identify novel diagnostic or therapeutic targets.

## Materials and methods

### Participants and data summary

In this study, we reanalyzed 85 oral metagenomic sequencing samples obtained from a publicly available cohort (**Supplementary Table S1**), which were downloaded from the European Bioinformatics Institute database under the accession code PRJNA289586. All diagnosed DM cases had a duration exceeding 5 years and were successfully managed with insulin replacement therapy, while the HC group exhibited no evidence of imminent DM risk based on autoantibody status.

Raw reads were preprocessed using fastp (v0.20.1) with the following parameters: -l 90 -q 20 -u 30 -y –trim_poly_g, to eliminate low-quality sequences. Quality-filtered reads were aligned to the human reference genome GRCh38 using bowtie2 (v2.4.1), and host-derived contaminants were subsequently identified and removed using default parameters.

### Oral virome database

To construct the oral virome reference database, we aggregated 2,792 publicly available oral metagenomic datasets from 16 global studies, encompassing samples collected from 12 distinct anatomical sites. These datasets were uniformly processed via a standardized bioinformatics pipeline, yielding 9.3 Tb of high-quality non-human metagenomic data. This pipeline culminated in an oral virome database (OVD) comprising 48,425 non-redundant vOTUs. The pipeline involved several key steps: (1) raw reads were quality-filtered and decontaminated from human sequences; (2) clean reads were assembled into contigs using SPAdes; (3) viral sequences were identified using CheckV (Nayfach et al., 2021), DeepVirFinder (Ren et al., 2020), and VIBRANT (Li et al., 2022), and decontaminated by removing those with high BUSCO ratios; and (4) viral sequences were clustered into viral operational taxonomic units (vOTUs) at 95% nucleotide identity across 75% of the sequence, with the longest sequence in each cluster serving as the representative. Specific data processing procedures were referenced from previous studies (Li et al., 2022).

### Metagenome-based oral virome profiling and analyses

To profile the oral viral community in fecal metagenomes of both DM patients and HCs, we utilized Bowtie 2 (v2.4.1) (Langmead, 2012) to align high-quality reads of all samples into the OVD. We set a nucleotide similarity threshold of 95% to define viral “species-level” taxa, following an established phylogenetic threshold (Gregory et al., 2019). By doing so, the abundance profile of vOTUs for each oral sample was produced by aggregating the number of reads mapped to each vOTU, and the relative abundances were generated by dividing it by the total mapped reads in that sample. We also produced a relative abundance profile at the viral family level by aggregating the relative abundances of vOTUs assigned to the same family.

We measured oral viral community richness using the observed number of vOTUs per family, while Shannon and Simpson indices were applied to assess virome diversity across all samples. These measures were computed using the *vegan* package (Dixon, 2003) in the R platform, with a consistent number of reads (20 million) for each sample.

### Oral bacteriome profiling

The oral microbial genome catalog was constructed from 3,569 prokaryotic species, encompassing over 50,000 metagenome-assembled genomes (MAGs), as reported in a previous study (Zhu et al., 2022). To analyze the bacterial communities in the oral metagenomes of DM patients and HC, high-quality reads from all samples were aligned against these MAGs using Bowtie 2 (v2.4.1). Phylum- and genus-level abundances were calculated through hierarchical aggregation of the proportions of constituent species.

### Construction of cross-kingdom co-occurrence network

Bacterial-vOTU interactions were analyzed using Spearman’s rank correlation with a stringent threshold (| r | > 0.8). Significant associations were visualized as interaction networks through Cytoscape v3.8.2 (Su et al., 2014).

### Statistical analysis

All statistical analyses in this study were conducted on the R (v4.2.1) platform. Principal coordinates analysis (PCoA) of the Bray-Curtis distance was carried out and visualized using the *vegan* package (Dixon, 2003). Permutational multivariate analysis of variance (PERMANOVA) was realized with the *adonis* function of the *vegan* package, with an *adonis p*-value generated from 1,000 permutations. We employed the Student’s t-test and Wilcoxon rank-sum test to assess statistical differences in the diversity and taxonomic levels between the two cohorts, respectively.

### Comparison of functional differences

In view of the functional differences between DM-enriched and HS-enriched vOTUs, the occurrence frequency of each KO in each group was first statistically analyzed. To test whether there is a significant difference in KO frequency between the two groups, Fisher’s exact test is performed through the fisher.test function. If the corrected p value is less than 0.05, it is determined that there is a significant functional difference of this KO between the two groups.

### Random forest modeling

To identify differential viral signatures distinguishing DM patients from HCs, we trained a Random Forest (RF) classifier using the *randomForest* package with 1,000 decision trees. RF is an ensemble learning algorithm that builds multiple decision trees and outputs either the majority vote (for classification) or the average prediction (for regression). In this study, the RF model was trained on high-dimensional, sparse vOTU abundance data, leveraging its robustness to such data structures. Feature importance was assessed via mean decrease in accuracy and Gini index, enabling implicit feature selection. To prevent overfitting, bootstrap aggregation (bagging) and random feature selection were employed. Model performance was evaluated using leave-one-out cross-validation (LOOCV), which iteratively trains the model on all samples except one and tests on the held-out sample, ensuring a rigorous assessment of predictive accuracy. We considered alternative models (for example, support vector machine, LASSO regression) but did not adopt them; RF is given priority due to its outstanding performance in dealing with nonlinear interactions and noise metagenomic characteristics.

## Results

### Diversity and structure of the oral virome in DM patients

**Figure 1:**
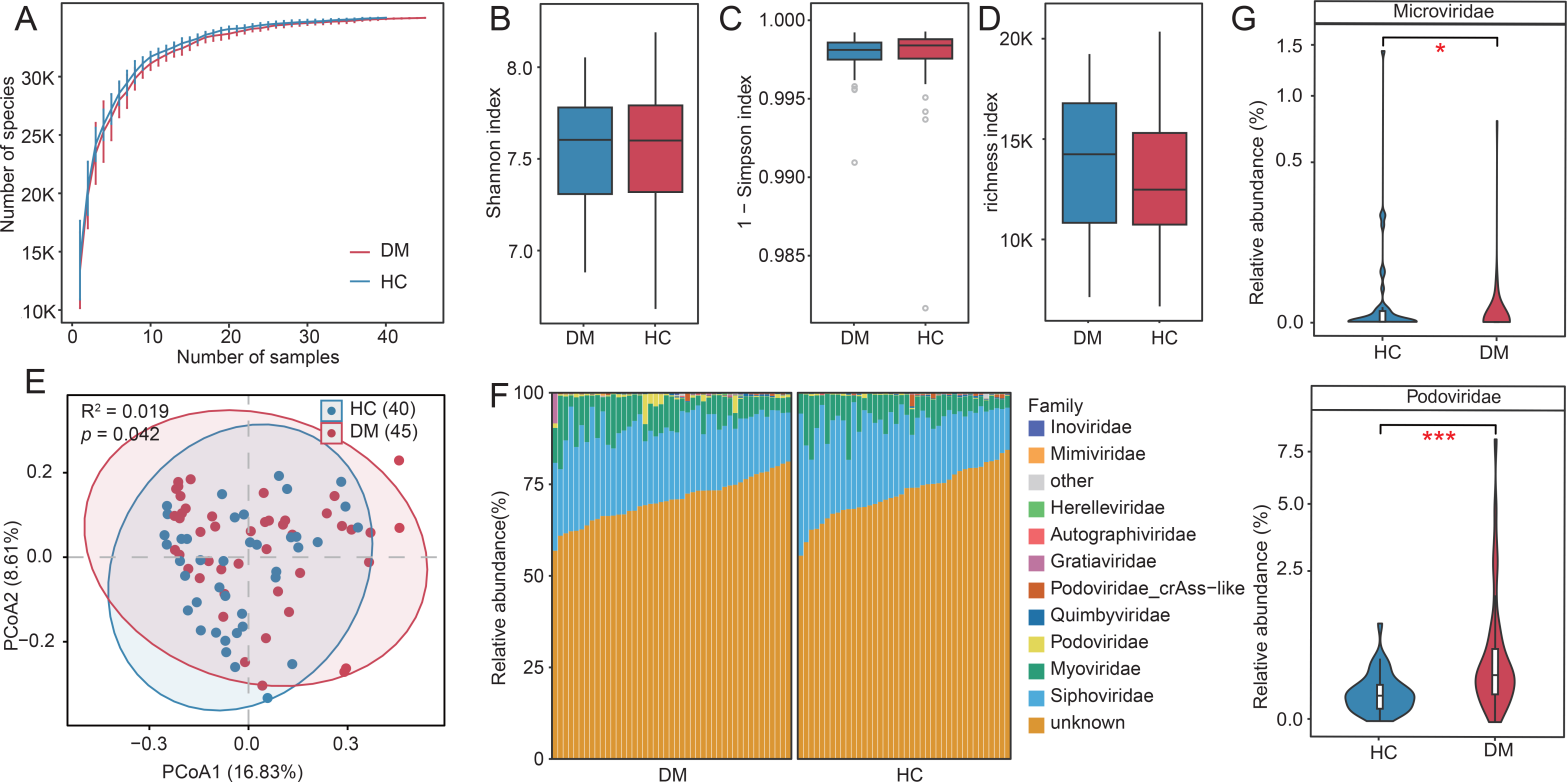
Diversity and compositional profiles of the oral virome in DM patients and HC. **(A)** Rarefaction curves of observed vOTUs across DM and HC groups. **(B-D)** Boxplots comparing α-diversity metrics between groups: Shannon index (left), Simpson index (middle), and richness index (right). **(E)** PCoA of β-diversity based on Bray-Curtis dissimilarity. Samples (nodes) are plotted along Principal Coordinates 1 (16.8% variance) and 2 (8.62% variance). Shaded ellipses represent 95% confidence intervals for group centroids. **(F)** Taxonomic composition of the oral virome at the family level (Top 10). **(G)** Violin plots highlighting differentially abundant viral families (*Podovirus*, *Microviridae*) between DM and HC cohorts (Wilcoxon test, p < 0.05). “*” p<0.05, “***”p<0.001.

To assess the impact of disease status on oral virome richness and evenness, we employed rarefaction curve analysis to quantify viral richness, measured as the number of observed vOTUs. Consistent with prior studies, no significant difference in viral richness was observed between DM patients and HC at equivalent sequencing depths (**Figure 1A**). Similarly, α-diversity metrics—including the Shannon, Simpson, and richness indices—showed no significant differences between the groups (Wilcoxon rank-sum test, p > 0.05; **Figures 1B–D**). For β-diversity analysis, we utilized Bray-Curtis dissimilarities at the vOTU level, incorporating PCoA and PERMANOVA. A significant separation between the DM and HC groups was observed (PERMANOVA: R² = 0.019, *p* = 0.042; **Figure 1E**). Taxonomic profiling at the family level revealed that annotated viral families constituted 29.31% of the total viral families. Specifically, the oral virome of DM patients was dominated by *Siphovirus* (mean relative abundance: 20.4%), *Myovirus* (7.2%), and *Podovirus* (0.86%). In contrast, HC individuals exhibited dominance of *Siphovirus* (20.16%), *Myovirus* (6.34%) and *Podovirus* (0.21%; **Figure 1F**). Notably, *Podovirus* (*p* = 0.00009*)* and *Microviridae* (*p* = *0.025)* showed statistically significant abundance differences between the cohorts (Wilcoxon rank-sum test; **Figure 1F**; **Supplementary Table S2**). *Podovirus* was enriched in DM patients, whereas *Microviridae* was more prevalent in HC groups. These findings suggest that dysbiosis in the oral virome may be linked to diabetes pathogenesis (**Figure 1G**).

### Identification of oral viral signatures associated with DM

**Figure 2:**
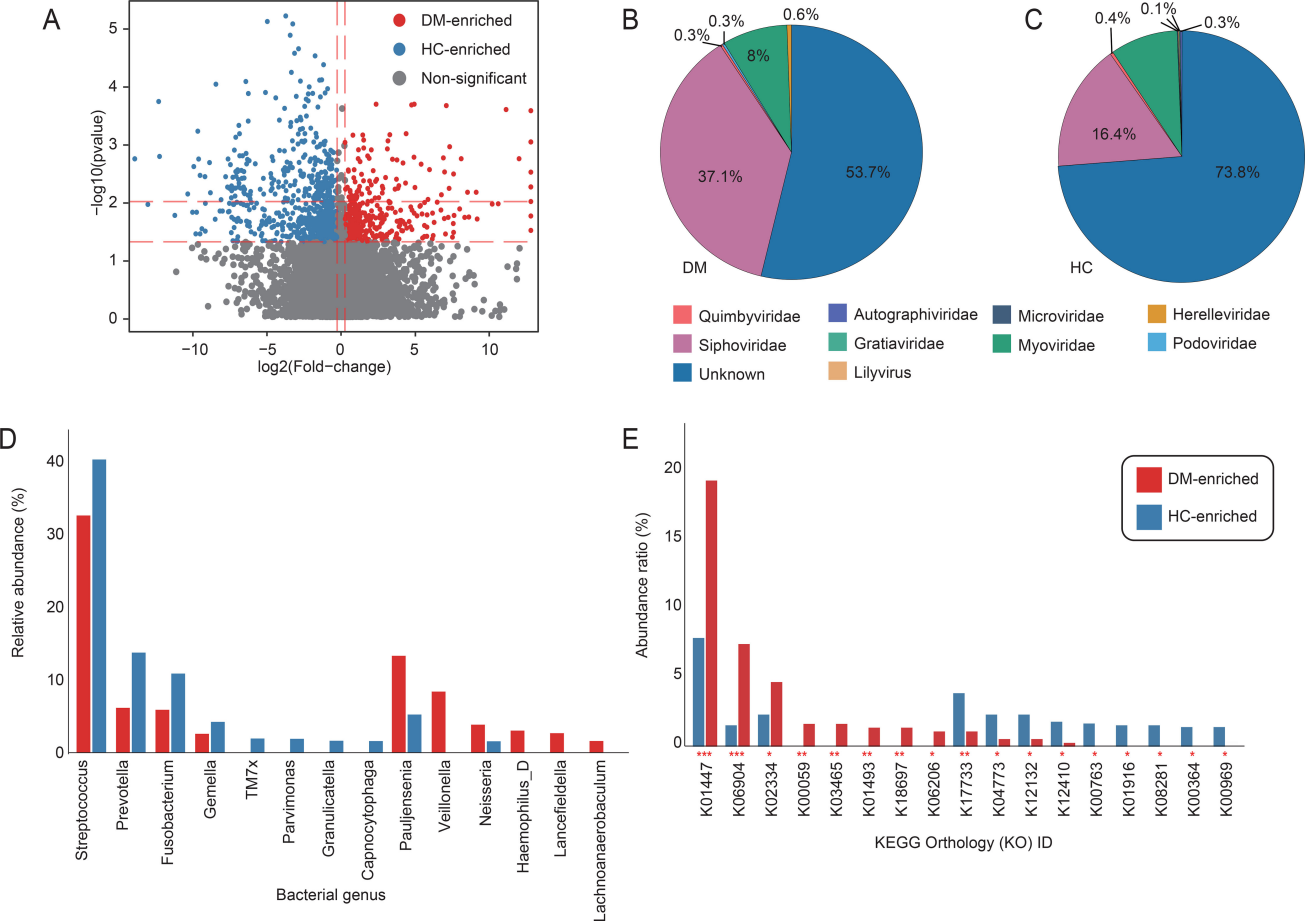
Viral taxonomic and host interaction signatures associated with DM. **(A)** Volcano plot of differential abundance analysis for vOTUs between DM patients and HC. Red dots denote vOTUs significantly enriched in DM (FC > 1.2; Benjamini-Hochberg-adjusted *q* < 0.05), while green dots indicate HC-enriched vOTUs (FC > 1.2; *q* < 0.05). Gray dots represent non-significant vOTUs. **(B, C)** Taxonomic distribution of DM-enriched and HC-enriched vOTUs at the viral family level. Sector sizes reflect the relative proportion of vOTUs assigned to each family. **(D)** Bar accumulation plots show the predicted host distributions of vOTUs enriched in the patient and control groups. **(E)** The functional differences of the oral virome between DM and HC. The red column represents the occurrence rate of the enriched KO pathway in the diabetes group; The blue column represents the incidence rate of the KO pathway enriched in the healthy control group. The vertical axis is the incidence rate of the pathway (%), and the horizontal axis is the number of the KO pathway.

Comparative analysis of the oral virome between DM patients and HC identified 1,131 vOTUs with significantly differential abundance (Wilcoxon rank-sum test, *p* < 0.05; **Figure 2A**; **Supplementary Tables S3**). Among these, 361 vOTUs were enriched in the DM patients, primarily affiliated with *Siphovirus* (37.1%), *Myovirus* (8%) and *Herelleviridae* (0.6%). In contrast, 770 HC-enriched vOTUs spanned seven families, dominated by Siphovirus (16.4%), *Myovirus* (8.8%) and *Quimbyviridae* (**Figures 2B, C**). We constructed a virus-host relationship based on genome homology alignment and CRISPR spacer sequence matching using established methods. The analysis revealed that DM-enriched vOTUs and HC-enriched vOTUs were primarily hosted by *Streptococcus* (>40%), followed by *Prevotella*, *Pauljensenia*, and others. However, we observed that HC-enriched vOTUs exhibited a stronger association with *Streptococcus*, *Prevotella*, *Fusobacterium* and *Gemella* species, while DM-enriched vOTUs were more closely linked to *Pauljensenia* and *Veillonella* bacteria. Notably, significant differences in vOTU signals were identified among DM patients (**Figure 2D**). Immediately after, we conducted a functional analysis of the differentiated votus and found that the enrichment patterns of different functional pathways in the DM group and the HC group were significantly different (**Figure 2E**; **Supplementary Table S4**). The proportion of K01447 et al. in DM-enriched was close to 20%, which was much higher than that in HC-enriched (about 7%), indicating that this functional pathway was highly enriched in the diabetes group and might be closely related to the pathophysiological process of diabetes. The enrichment of K17733 et al. was more prominent in the HC group, reflecting that this functional pathway was more active in the healthy control group and perhaps an important pathway for maintaining normal physiological states.

### Oral cross-kingdom co-occurrence network analysis in DM patients

**Figure 3:**
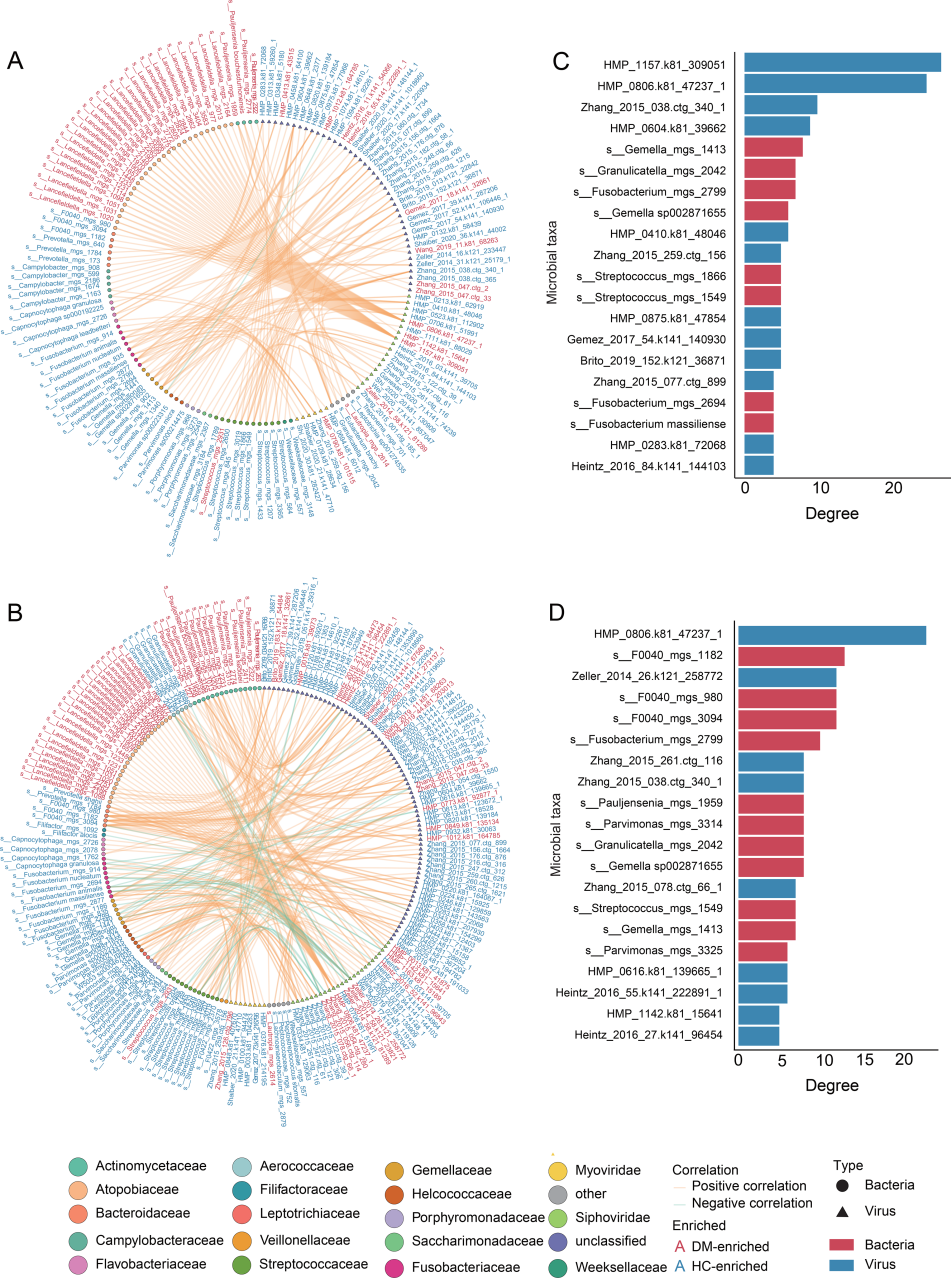
Cross-kingdom co-abundance networks and core taxa in DM patients and HC network. **(A, B)** Spearman correlation networks between viral and bacterial signatures in DM patients **(A)** and HC **(B)**. Node colors represent viral or bacterial classification at the family level, with triangular nodes denoting viruses and circular nodes representing bacterial taxa. Orange edges indicate positive correlations, while green edges represent negative correlations. Bacterial names highlighted in red denote taxa enriched in DM patients, whereas those in blue represent taxa significantly depleted in DM patients. **(C, D)** Bar charts ranking the top 20 virus-bacteria pairs by correlation strength in **(C)** DM and **(D)** HC. Blue bars represent viruses, while red bars denote bacteria.

Using an analytical approach analogous to our virome methodology, we identified 195 differential bacterial species between DM patients and HCs. To investigate cross-kingdom interactions between DM-associated oral viral and bacterial signatures, we performed Spearman’s rank correlation analysis on 1,131 viral signatures and 195 bacterial signatures (**Supplementary Table S5**). Applying a stringent threshold (|r| > 0.8, p < 0.05), ​we identified 189 significant virus-bacteria associations in DM patients (**Figure 3A**) and 209 in HC (**Figure 3B**). The two networks exhibited distinct interaction patterns. Specifically, the DM network was predominantly composed of positive correlations, indicative of a relatively unstable network structure (Coyte et al., 2015). Further analysis of network hub nodes revealed that, in the DM network, a specific viral species (*HMP_1157.k81_309051*) played a central role, influencing multiple bacterial taxa (e.g., *Lancefieldella*, *Pauljensenia*, **Figure 3C**). In contrast, the HC network highlighted the potential importance of the bacterial genus *F0040* (**Figure 3D**), which appeared to play a key role in maintaining healthy oral interactions. Notably, this relationship was decrease in the DM network, suggesting that *F0040* may be crucial for sustaining healthy oral microbial interactions.

### Diagnostic potential of oral virome and bacteriome in DM

**Figure 4:**
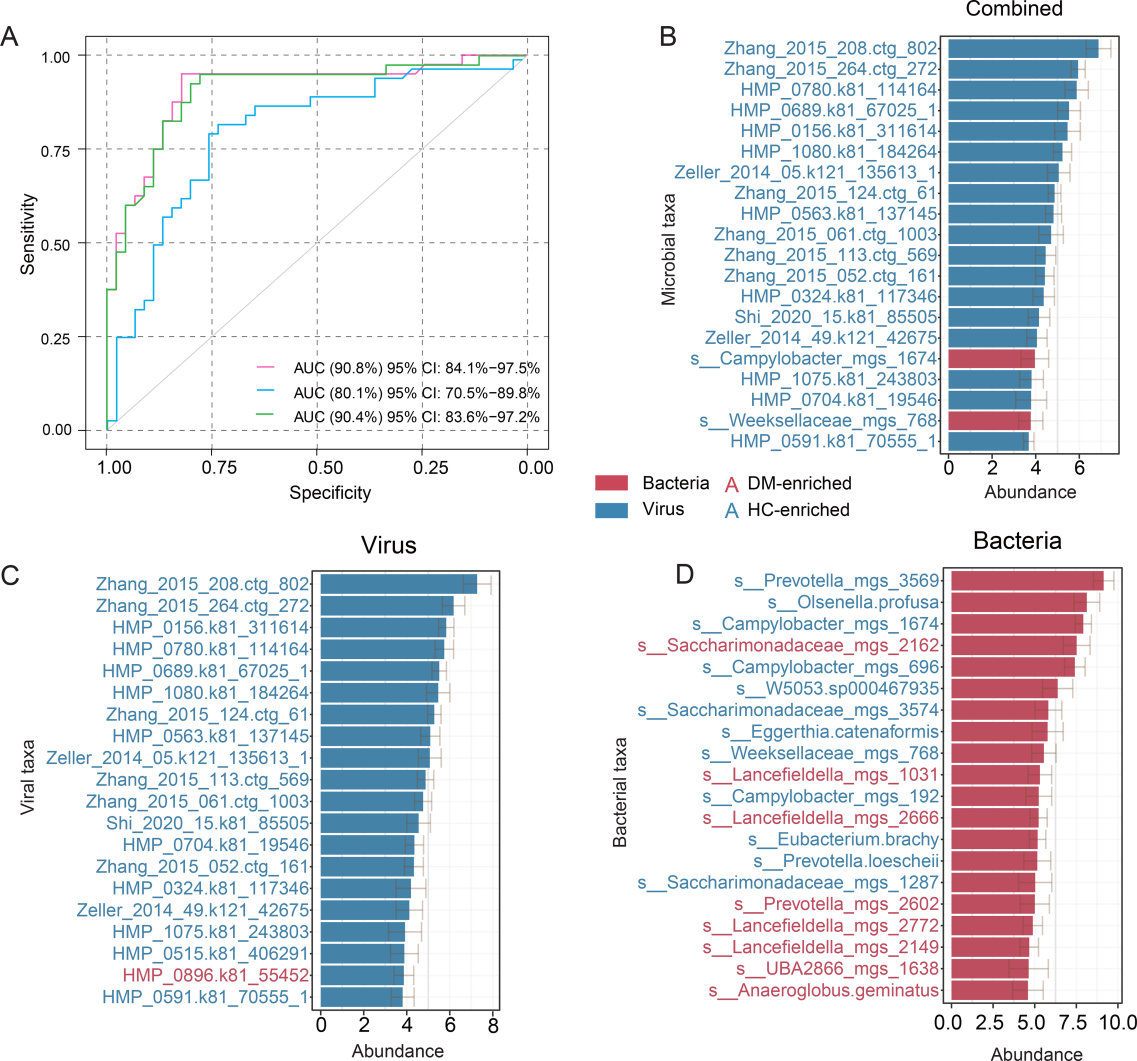
Classification of DM status based on the abundances of the oral bacteriome and virome. **(A)** ROC analysis for DM classification using bacterial and viral signatures derived from a random forest model. **(B–D)** The top 20 most discriminant microbial signatures identified by the random forest model for the combined approach **(B)**, virome **(C)**, and bacteriome **(D)**. Bar lengths represent the mean variable importance across multiple iterations, with error bars indicating the standard deviation to reflect variability in feature ranking. Red bars denote taxa enriched in DM patients, while blue bars indicate taxa depleted in DM patients.

To evaluate the diagnostic potential of oral microbiome features for DM classification, we trained random forest models using three distinct feature sets: bacteriome, virome, and a combined (bacteriome-virome) approach. The virome model demonstrated the highest diagnostic accuracy, achieving an area under the receiver operating characteristic curve (AUC) of 90.8% (95% CI: 84.1 – 97.5%). In comparison, the bacteriome model yielded a lower AUC of 80.1% (95% CI: 70.5 – 89.8%), while the combined model exhibited performance comparable to the virome model (AUC = 90.4%, 95% CI: 83.6 – 97.2%) (**Figure 4A**). Furthermore, by analyzing feature importance across these models, we consistently observed that viral features held greater significance in the diagnostic framework (**Figures 4B–D**). Collectively, these findings highlight the potential of oral viral signatures as valuable biomarkers for DM diagnosis, offering a promising avenue for future microbiome-based diagnostic strategies.

## Discussion

The global rise in DM, driven by aging populations and environmental factors, has established it as a leading endocrine-metabolic disorder worldwide. While a strong bidirectional relationship between oral microbial dysbiosis and DM pathogenesis has been documented (Sanz et al., 2018; Rohani, 2019), the role of the virome in DM remains poorly characterized. In this study, we analyzed the oral virome of a cohort comprising 85 individuals (DM patients vs. HC group) to evaluate viral diversity, taxonomic composition, and cross-kingdom network shifts. Specifically, although no significant differences in viral α-diversity were observed between groups, a large number of viral signatures revealed significant alterations in the oral virome of DM patients, demonstrating strong predictive performance in disease diagnosis. Furthermore, cross-kingdom network analysis demonstrated unique microbial interactions in the oral microbiome of DM patients.

At the family level, compositional profiling revealed significant enrichment of *Podovirus* in the DM group, which aligns with previous findings in severe early childhood caries (Chen et al., 2024). In diabetes patients, oral inflammation (e.g., periodontitis) leads to suboptimal glycemic control, a higher incidence of diabetes-related complications, and conversely, poor blood sugar management exacerbates oral microbial pathogenicity, forming a vicious cycle (Graziani et al., 2018; Diao et al., 2024). By contrast, *Microviridae* was more abundant in the HC group. Studies have shown that *Microviridae* are small phages that infect Gram-negative commensal bacteria. They may protect microenvironment homeostasis by modulating bacterial population balance, suppressing overgrowth of opportunistic pathogens, and sustaining the metabolic activity of short-chain fatty acid-producing bacteria (Ríos-Covián et al., 2016; Lekunberri et al., 2017; Gogokhia et al., 2019). The distinct enrichment patterns of *Podovirus* and *Microviridae* suggest their divergent ecological roles in the diabetic oral niche. *Podovirus* primarily infecting Gram-positive bacteria, likely target key oral pathogens such as Streptococcus and Actinomyces (Kövér et al., 2023)– consistent with our observations of DM-enriched Streptococcus and *Pauljensenia* (*Actinomyces*) phages. In diabetic individuals, chronic hyperglycemia may promote lytic activation of these phages, exacerbating tissue damage through bacterial lysis-induced inflammation (Giri et al., 2018; Xu et al., 2022). Conversely, *Microviridae’s* preference for Gram-negative commensals (e.g., *Prevotella*, *Fusobacterium*) positions them as potential homeostasis modulators (Krupovic and Forterre, 2011). Their reduction in DM could permit overgrowth of LPS-producing bacteria, impairing epithelial barrier function and fueling metabolic endotoxemia (Fan et al., 2023). These findings highlight the intricate interplay between viral communities, oral and systemic health, and metabolic regulation, emphasizing the need for further research to elucidate these complex interactions.

We identified numerous DM-associated vOTUs and observed that DM-enriched viruses were predominantly composed of *Streptococcus*, *Pauljensenia*, and *Veillonella* phages, whereas HC-enriched vOTUs were dominated by *Prevotella*, *Fusobacterium*, and *Gemella*. It is plausible that the association between Gemella-infecting bacteriophages—enriched in healthy controls—and Gemella itself may be linked to butyric acid production. This connection, although speculative, suggests a potential role for these phages in supporting intestinal barrier integrity and limiting LPS translocation through modulation of butyrate-producing pathways (Arpaia et al., 2013; Byndloss et al., 2017; Zhang and Wang, 2023). K01447 (N-acetylmuramyl-L-alanine amidase) was significantly enriched in the DM group. This enzyme hydrolyzes bacterial cell wall peptidoglycan to release muramyl-dipeptide (MDP), which activates the host’s innate immune receptor NOD2 and drives chronic inflammatory responses (Negroni et al., 2018). Importantly, current evidence suggests that this process may operate synergistically with *Veillonella* phage-mediated excessive Th17 activation. This potential synergy could contribute to IL - 17A/IL-22 releases, which may further exacerbate insulin resistance and systemic inflammation (Korn et al., 2008; Talukdar et al., 2012; Smith et al., 2013). K17733 (peptidoglycan LD-endopeptidase CwlK) was highly active in the HC group. It functions to maintain the structural stability of the flora by precisely regulating peptidoglycan hydrolysis.

Microbial interaction network analysis in this study further revealed that the restructuring of the diabetic oral ecosystem is characterized not only by shifts in viral abundance but also by a dynamic imbalance in virus–bacteria synergistic relationships. Although the total number of co-abundance relationships was similar between the HC and DM groups, the feature of viral nodes occupying key hub positions was particularly prominent in the DM group. In the bacterial-viral correlation analysis of the oral microbiome, the virus *HMP_0806.k81_47237_1* maintained high connectivity (degree > 20) in both the HC and DM groups, indicating its critical role in maintaining oral homeostasis. By contrast, the association strength of *HMP_1157.k81_309051* increased significantly in DM, suggesting that under specific environmental pressures (e.g., DM microenvironment), this virus may exacerbate microbial dysbiosis and host inflammation. Notably, *F0040* (bacterial genus) played a central role as a core species in the HC network, yet its potential function in the oral cavity remains underexplored in diabetes-related studies, despite its observed associations with *Veillonella* and *Streptococcus* in other research contexts (Liu et al., 2023). Moreover, in the construction of diagnostic models, viral community features outperformed bacterial features in predictive accuracy. This challenges the conventional bacteria-centric paradigm in microbiome-based diagnostics and highlights the potential of the oral virome as a novel biomarker and early warning indicator for diabetes. These findings offer new insights and directions for the early detection and intervention of diabetes through virome-based approaches. It is worth noting that the current interaction network is constructed based on Spearman correlations, which capture statistical associations but do not imply causality. Although this method is widely used to reveal co-abundance patterns in microbiome research, it does not reflect the directionality or underlying mechanisms of virus–bacteria interactions. Therefore, future studies should consider incorporating longitudinal sampling and causal inference techniques—such as Granger causality, structural equation modeling, or dynamic Bayesian networks—to more precisely elucidate the direction and impact of these microbial interactions under disease conditions.

However, this study has limitations that warrant consideration. ​First, the moderate sample size (n = 85) may constrain the statistical power and generalizability of our findings. Although compelling, these results necessitate validation in larger, multi-center cohorts to confirm their robustness. Second, the absence of longitudinal or prospective data ​limits our ability to infer causal relationships between oral virome dynamics and DM pathogenesis. Third, critical confounding variables—including periodontal status, oral hygiene habits, medication use (e.g., antibiotics or antidiabetic drugs), and detailed glycemic control indicators (e.g., HbA1c levels)—were unavailable in the original public dataset (PRJNA289586). This limitation was unavoidable due to the metadata constraints of the source data, and may bias the observed associations between virome alterations and diabetes. Additionally, current taxonomic ambiguities in oral virus classification hinder mechanistic exploration. Thus, integrating animal models is essential to elucidate potential pathogenic mechanisms linking oral virome to DM.

## Conclusion

In summary, through metagenomic sequencing of oral samples from DM patients, we characterized the oral virome alterations in DM, identifying 1,131 DM-associated viral signatures and disrupted cross-kingdom interactions. Notably, ​virus-based classifiers achieved high accuracy (AUC = 90.8%) in predicting DM status, highlighting translational potential. Critically, our finding that DM-associated virome alterations center on bacteriophage families (*Podovirus*/*Microviridae*) with opposing impacts on bacterial dysregulation reveals an underappreciated viral layer in diabetes pathology. Nevertheless, ​the moderate cohort size (n = 85) and lack of external validation require caution in generalizing these findings. Future studies must verify these results in independent, expanded cohorts and address causal links through longitudinal designs. Our work provides a foundation for mechanistic and clinical studies on oral virome-DM interplay.

## Data Availability

The original contributions presented in the study are included in the article/**Supplementary Material**. Further inquiries can be directed to the corresponding authors.
